# Supplementary material for: H2O2 Induces Association of RCA with the Thylakoid Membrane to Enhance Resistance of Oryza meyeriana to Xanthomonas oryzae pv. oryzae
Source: Plants (Basel). 2019 Sep 16;8(9):351. doi: 10.3390/plants8090351 (PMC6784163; doi:10.3390/plants8090351)
Supplement: Supplementary file 1 [file plants-08-00351-s001.pdf]

Supplementary Table 1. Effects of *Xoo* inoculation on *Fv*/*Fm*, Φ_{PSII}, *qP* and *qN* in the leaves of *O. meyeriana* and *O. sativa* during a 336 h test period. Attached leaves were kept in the dark for adaptation for 1 h before the measurement of chlorophyll fluorescence. *Fv*/*Fm*: PSII maximum photochemical efficiency; Φ_{PSII}: PSII effective photochemical efficiency; *qP*: PSII photochemical quenching; *qN*: PSII non-photochemical quenching. Results are the means ± standard error of three replications.

Time after inoculation (h)	*O. meyeriana*				*O. sativa*			
	Fv/*Fm*	Φ_{PSII}	*qP*	*qN*	*Fv*/*Fm*	Φ_{PSII}	*qP*	*qN*
0	0.782 ± 0.002	0.597 ± 0.014	0.776 ± 0.009	0.343 ± 0.011	0.784 ± 0.015	0.597 ± 0.014	0.779 ± 0.013	0.346 ± 0.015
1	0.785 ± 0.010	0.596 ± 0.008	0.781 ± 0.009	0.344 ± 0.010	0.780 ± 0.010	0.596 ± 0.014	0.784 ± 0.006	0.348 ± 0.018
2	0.785 ± 0.012	0.606 ± 0.008	0.780 ± 0.013	0.357 ± 0.010	0.780 ± 0.015	0606. ± 0.011	0.783 ± 0.009	0.360 ± 0.011
3	0.787 ± 0.007	0.607 ± 0.011	0.778 ± 0.008	0.359 ± 0.013	0.779 ± 0.012	0.607 ± 0.010	0.781 ± 0.009	0.362 ± 0.015
4	0.782 ± 0.002	0.589 ± 0.010	0.780 ± 0.016	0.355 ± 0.011	0.783 ± 0.018	0.589 ± 0.010	0.783 ± 0.019	0.359 ± 0.012
5	0.786 ± 0.008	0.598 ± 0.008	0.783 ± 0.009	0.368 ± 0.009	0.785 ± 0.012	0.598 ± 0.008	0.786 ± 0.019	0.371 ± 0.017
6	0.787 ± 0.009	0.589 ± 0.004	0.783 ± 0.012	0.367 ± 0.018	0.781 ± 0.010	0.589 ± 0.014	0.786 ± 0.013	0.370 ± 0.012
7	0.785 ± 0.010	0.596 ± 0.006	0.785 ± 0.013	0.369 ± 0.016	0.785 ± 0.012	0.596 ± 0.008	0.789 ± 0.015	0.373 ± 0.016
8	0.782 ± 0.002	0.603 ± 0.007	0.777 ± 0.017	0.363 ± 0.015	0.785 ± 0.013	0.597 ± 0.008	0.781 ± 0.012	0.366 ± 0.013
9	0.785 ± 0.010	0.586 ± 0.006	0.767 ± 0.008	0.378 ± 0.014	0.786 ± 0.009	0.589 ± 0.022	0.773 ± 0.016	0.381 ± 0.013
10	0.785 ± 0.012	0.572 ± 0.003	0.759 ± 0.008	0.407 ± 0.013	0.782 ± 0.015	0.589 ± 0.014	0.774 ± 0.017	0.387 ± 0.011
11	0.786 ± 0.009	0.561 ± 0.002	0.736 ± 0.005	0.453 ± 0.014	0.781 ± 0.015	0.592 ± 0.016	0.781 ± 0.009	0.387 ± 0.012
12	0.783 ± 0.001	0.524 ± 0.002	0.692 ± 0.010	0.535 ± 0.008	0.781 ± 0.015	0.581 ± 0.007	0.775 ± 0.014	0.388 ± 0.011
13	0.785 ± 0.010	0.467 ± 0.005	0.622 ± 0.005	0.599 ± 0.015	0.782 ± 0.017	0.595 ± 0.017	0.776 ± 0.011	0.397 ± 0.009
14	0.786 ± 0.010	0.493 ± 0.004	0.648 ± 0.009	0.565 ± 0.011	0.784 ± 0.012	0.589 ± 0.011	0.771 ± 0.006	0.388 ± 0.011
15	0.784 ± 0.012	0.525 ± 0.004	0.686 ± 0.006	0.545 ± 0.012	0.782 ± 0.017	0.595 ± 0.014	0.772 ± 0.014	0.395 ± 0.016
16	0.783 ± 0.001	0.546 ± 0.004	0.710 ± 0.008	0.504 ± 0.010	0.787 ± 0.017	0.601 ± 0.012	0.773 ± 0.008	0.395 ± 0.014
17	0.786 ± 0.009	0.566 ± 0.005	0.743 ± 0.013	0.465 ± 0.011	0.784 ± 0.007	0.590 ± 0.008	0.771 ± 0.017	0.397 ± 0.019
18	0.786 ± 0.011	0.581 ± 0.006	0.759 ± 0.009	0.424 ± 0.011	0.785 ± 0.014	0.591 ± 0.014	0.770 ± 0.010	0.397 ± 0.019
19	0.788 ± 0.006	0.601 ± 0.006	0.768 ± 0.006	0.398 ± 0.013	0.774 ± 0.014	0.587 ± 0.014	0.769 ± 0.009	0.398 ± 0.016
20	0.786 ± 0.004	0.589 ± 0.003	0.770 ± 0.011	0.394 ± 0.009	0.777 ± 0.015	0.584 ± 0.017	0.768 ± 0.012	0.401 ± 0.010
21	0.785 ± 0.010	0.595 ± 0.006	0.771 ± 0.012	0.395 ± 0.019	0.774 ± 0.008	0.581 ± 0.011	0.763 ± 0.021	0.387 ± 0.017
22	0.791 ± 0.002	0.601 ± 0.004	0.778 ± 0.008	0.393 ± 0.011	0.774 ± 0.014	0.577 ± 0.008	0.762 ± 0.011	0.371 ± 0.015
23	0.788 ± 0.003	0.596 ± 0.007	0.772 ± 0.012	0.392 ± 0.012	0.762 ± 0.009	0.571 ± 0.008	0.747 ± 0.017	0.358 ± 0.009
24	0.782 ± 0.003	0.598 ± 0.006	0.773 ± 0.010	0.383 ± 0.009	0.747 ± 0.005	0.567 ± 0.013	0.739 ± 0.008	0.357 ± 0.009
25	0.785 ± 0.007	0.597 ± 0.006	0.768 ± 0.009	0.384 ± 0.019	0.742 ± 0.012	0.556 ± 0.012	0.713 ± 0.008	0.335 ± 0.011
26	0.786 ± 0.009	0.599 ± 0.011	0.768 ± 0.012	0.385 ± 0.009	0.722 ± 0.010	0.544 ± 0.019	0.668 ± 0.006	0.328 ± 0.010
72	0.784 ± 0.007	0.597 ± 0.007	0.770 ± 0.004	0.383 ± 0.008	0.629 ± 0.017	0.486 ± 0.013	0.541 ± 0.018	0.274 ± 0.008
168	0.785 ± 0.007	0.593 ± 0.004	0.766 ± 0.007	0.377 ± 0.010	0.438 ± 0.013	0.293 ± 0.006	0.352 ± 0.017	0.168 ± 0.014
336	0.790 ± 0.003	0.596 ± 0.007	0.765 ± 0.009	0.382 ± 0.015	0.332 ± 0.018	0.167 ± 0.012	0.225 ± 0.013	0.022 ± 0.012
